# Supplementary material for: TSSC4 is a component of U5 snRNP that promotes tri-snRNP formation
Source: Nat Commun. 2021 Jun 15;12:3646. doi: 10.1038/s41467-021-23934-y (PMC8206348; doi:10.1038/s41467-021-23934-y)
Supplement: Supplementary file 1 — Supplementary_information [file 41467_2021_23934_MOESM1_ESM.pdf]

SUPPLEMENTARY INFORMATION

TSSC4 is a component of U5 snRNP that promotes tri-snRNP formation

Klára Klimešová et al.

Supplementary Figure 1

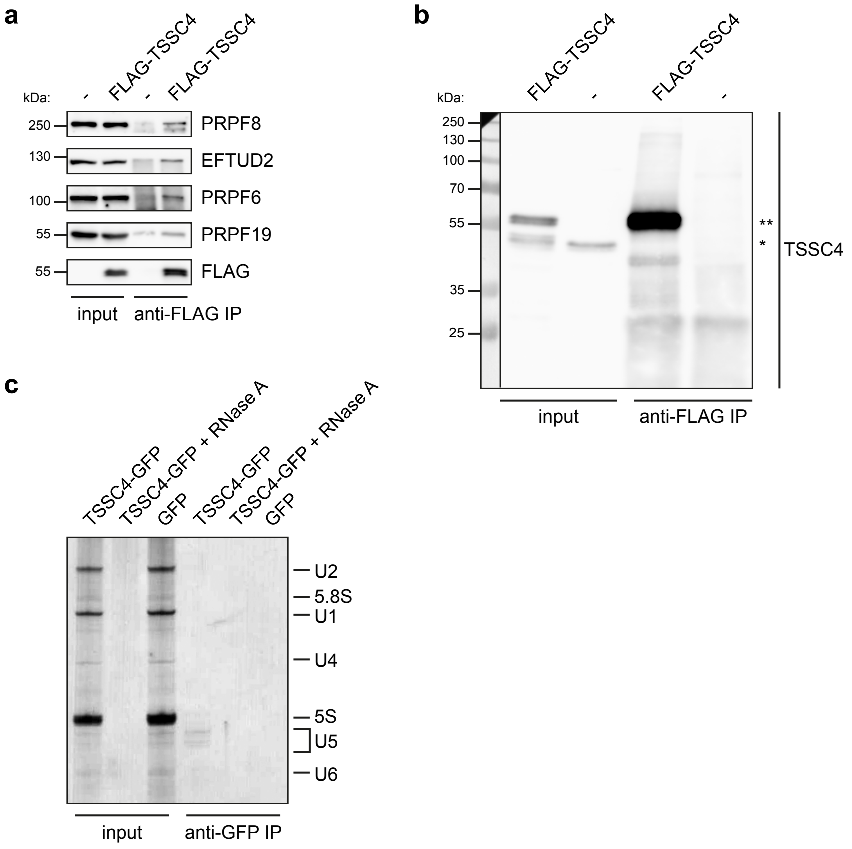

Supplementary Figure 1. TSSC4-FLAG co-precipitates U5 snRNP proteins and does not self-interact.
a TSSC4-FLAG immunoprecipitation followed by Western blotting and detection of proteins specific for U5 (PRPF8, EFTUD2 and PRPF6) and PRPF19 particles (PRPF19). The positions of molecular weight markers are shown. **b** TSSC4-FLAG immunoprecipitation followed by Western blotting and detection with anti-TSSC4 (left panel) and anti-FLAG (right panel) antibodies. Endogenous TSSC4 is marked by *, TSSC4-FLAG by **. Molecular weight marker is shown on the left side. **c** Cell extracts were treated with RNAse A followed by TSSC4-GFP immunoprecipitation and analysis of co-precipitated snRNAs by silver staining in denaturing urea polyacrylamide gels. **a-c** The experiments were performed once. Uncropped and unprocessed scans are provided in the Source Data file.

Supplementary Figure 2

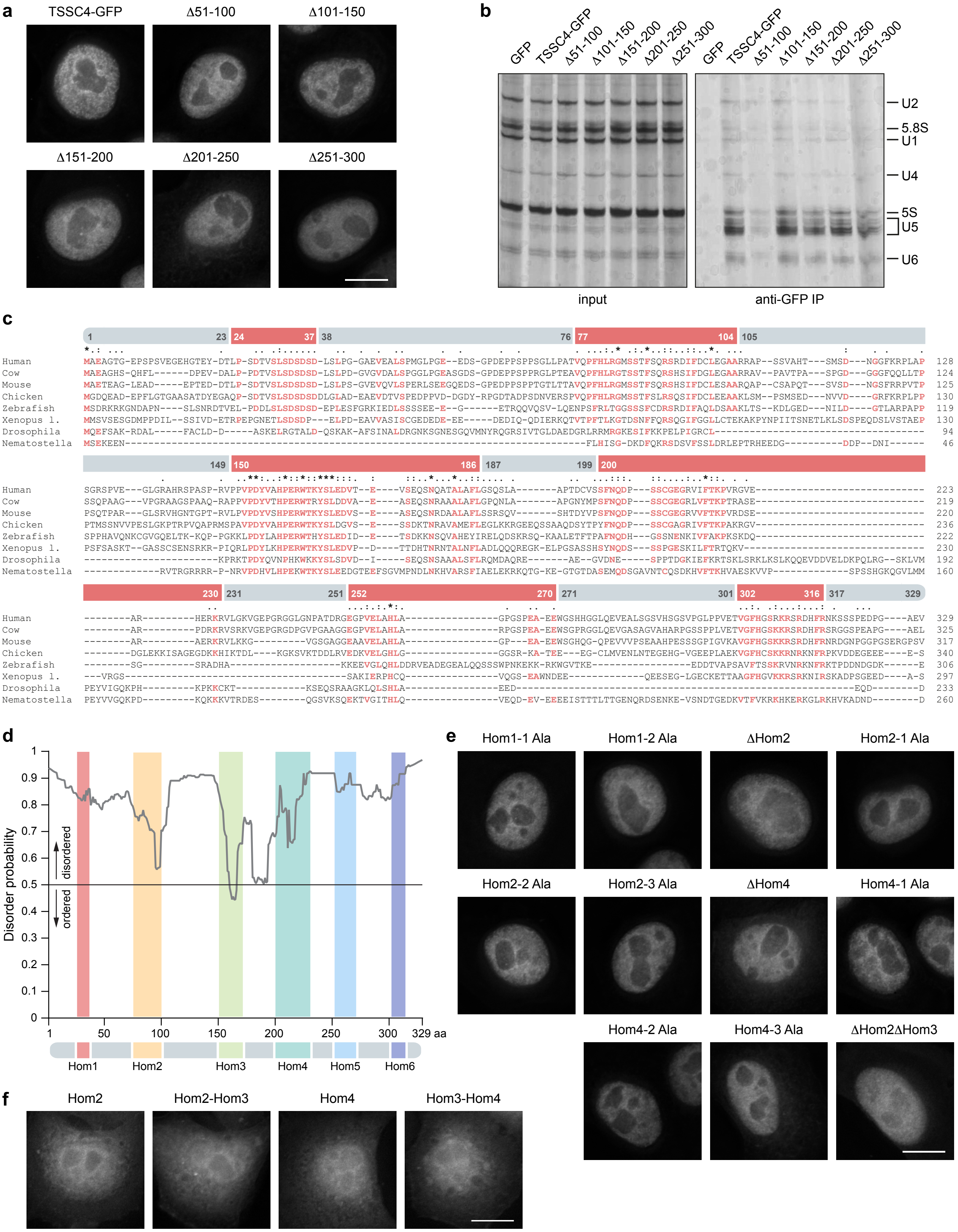

Supplementary Figure 2. Localization of TSSC4 mutants in the cell and their interaction with snRNAs.
a Individual TSSC4 deletion mutants were tagged with GFP and transiently expressed in HeLa cells. Scale bar 10μm. **b** Co-immunoprecipitation of U5 snRNA with TSSC4 deletion mutants. TSSC4-GFP full-length or individual deletion construct were transiently expressed in HeLa cells and immunoprecipitated by anti-GFP antibodies. Co-precipitated RNAs were isolated, resolved on a denaturing UREA polyacrylamide gel and silver-stained. The experiment was performed once.Uncropped and unprocessed scan is provided in the Source Data file. **c** Homology alignment of TSSC4 done using T-Coffee tool. Used sequences were downloaded from Uniprot database and are based either on experimental evidence of TSSC4 protein (human, mouse, Xenopus), mRNA (cow, chicken, zebrafish, Drosophila) or protein prediction (Nematostella). Amino acids with more than 65% identity are highlighted in red. **d** Probability of TSSC4 to adopt disordered structures. TSSC4 sequence was analyzed by MetaDisorderMD2 to predict a probability to take up disrodered structure. Original values for individual amino acids are provided in the Source Data file **e,f** TSSC4 mutants were tagged with GFP and transiently expressed in HeLa cells. Scale bar 10μm. The experiments were performed once with at least 6 cells analyzed for each mutant in a, e and f.

Supplementary Figure 3

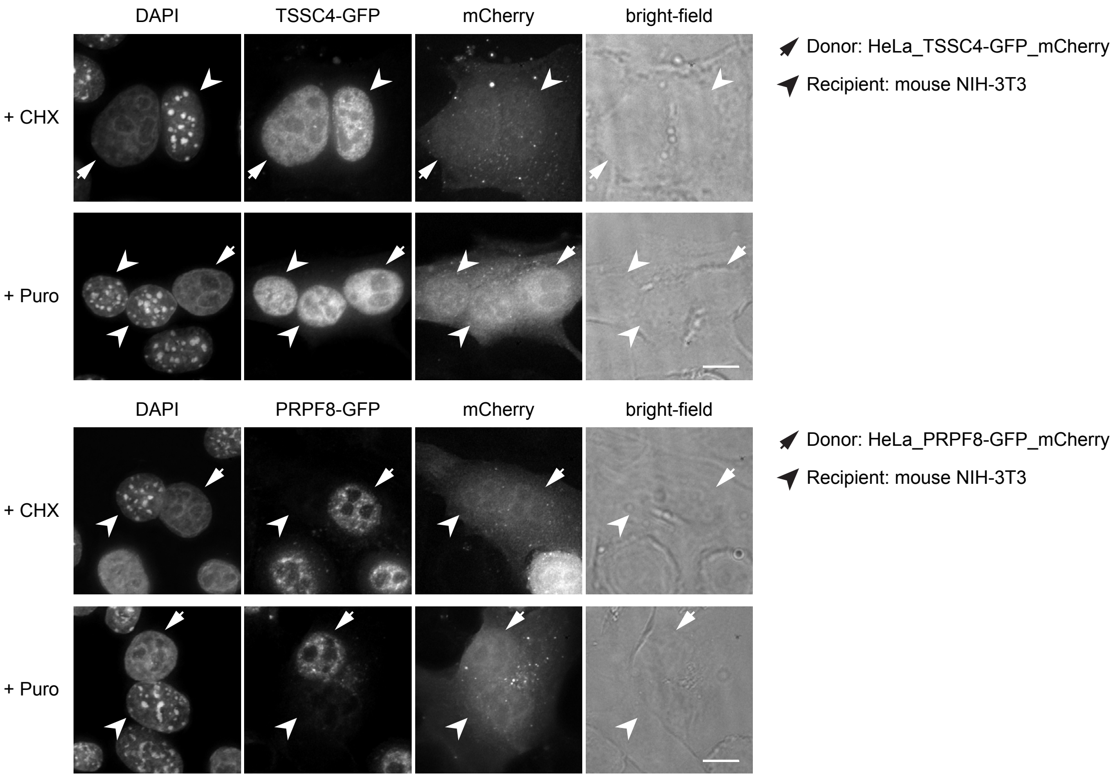

Supplementary Figure 3. TSSC4 shuttles between the nucleus and the cytoplasm. TSSC4-GFP or PRPF8-GFP were expressed together with mCherry in HeLa cells (arrow). After protein synthesis inhibition by cycloheximide (CHX) or Puromycin (Puro), HeLa cells were fused by PEG treatment with mouse NIH-3T3 fibroblasts (arrowhead). Cells were incubated for three hours and the presence of mCherry as a positive control and GFP signal in mouse nuclei was monitored. TSSC4-GFP clearly localized to recepient mouse nuclei identified by distinct DAPI staining while PRPF8-GFP, which does not cycle stayed accumulated in HeLa nuclei only. Scale bar 10μm.The experiment was performed in two biological replicates and in every replicate at least 5 different heterokaryons were analyzed with similar results for each condition.

Supplementary Figure 4

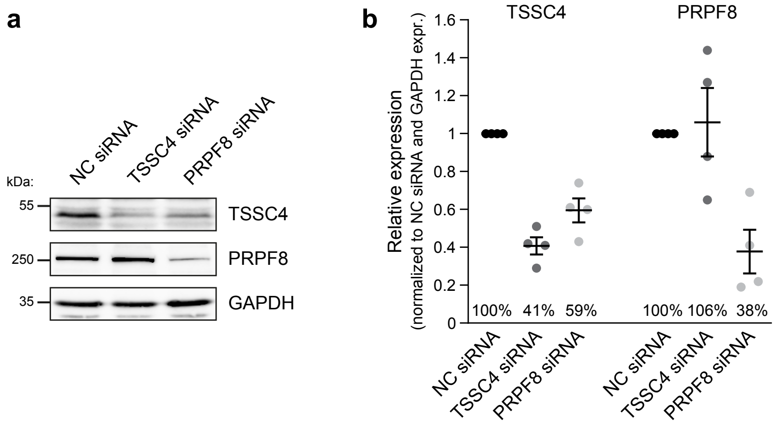

Supplementary Figure 4. Knockdown efficiency of TSSC4 and PRPF8. a HeLa cells were treated with 50nM siRNA for 72h. Proteins were then isolated and detected by Western blotting. The positions of molecular weight markers are shown. Uncropped and unprocessed scans are provided in the Source Data file. **b** Quantification of TSSC4 and PRPF8 knockdown efficiency in four biological replicates. TSSC4 and PRPF8 signal was normalized to GAPDH. Mean and SEM are shown and the exact value of the mean calculated as a percentage of negative control is written at the bottom of the diagram. Values for graphs are provided in the Source Data file.

Supplementary Figure 5

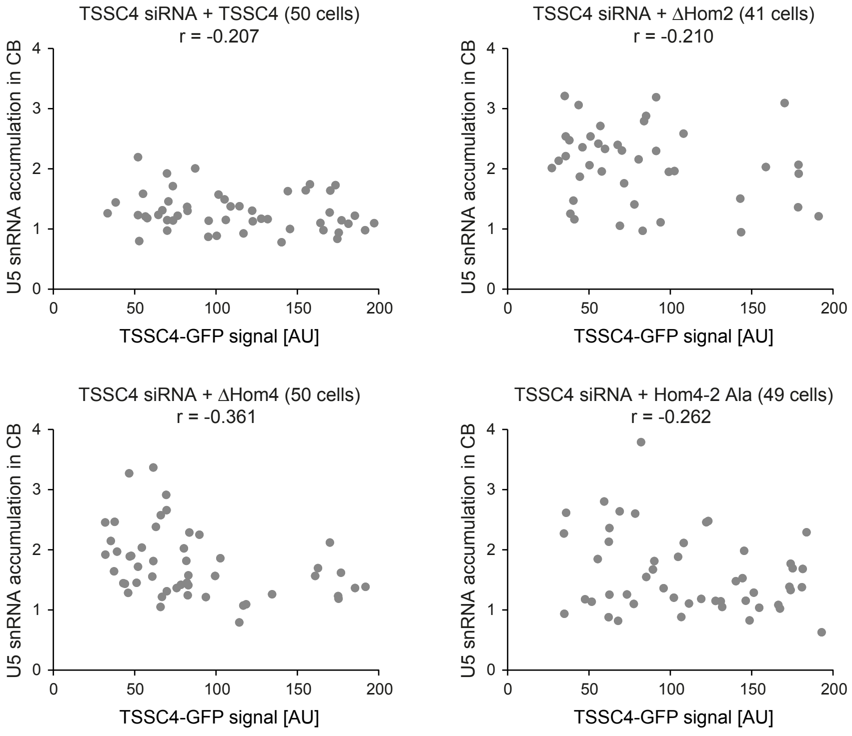

Supplementary Figure 5. Relationship between U5 snRNA Cajal body accumulation and TSSC4-GFP expression. In-situ hybridization signal in the Cajal body was normalized to the average signal in the nucleoplasm. The normalized U5 snRNA accumulation in individual cells is plotted together with TSSC4-GFP expression. The scale for GFP signal was set between 0 and 256 and cells with GFP signal between 25-200 were selected for the measurements. Pearson correlation was calculated in Excel and showed no correlation between U5 snRNA Cajal body accumulation and TSSC4-GFP expression for three out of four constructs. A very weak negative correlation was observed for the ΔHom4 construct. Values for graphs are provided in the Source Data file.

Supplementary Table 1. Results of yeast two-hybrid assay

Interaction of TSSC4 with U5 specific proteins and PRPF19

Amino acids	ActII:	As2DD:		As2DD:	ActII:		
		TSSC4			TSSC4	Alix (NC)	
	Alix (NC)	-					
1-387	PRPF8 A	-		PRPF8 A	-	-	
385-818	PRPF8 B	-		PRPF8 B	-	-	
818-1259	PRPF8 C	-		PRPF8 C	-	-	
1259-1775	PRPF8 D	-		PRPF8 D	-	-	
1775-2035	PRPF8 E	-		PRPF8 E	-	-	
2035-2335	PRPF8 F	-		PRPF8 F	-	-	
1-434	SNRNP200 A	-		SNRNP200 A *	-	-	* 5 mM 3-AT
393-832	SNRNP200 B	-		SNRNP200 B	-	-	
807-1296	SNRNP200 C	-		SNRNP200 C	-	-	
1296-1818	SNRNP200 D	-		SNRNP200 D *	-	-	* 5 mM 3-AT
1809-2136	SNRNP200 E	-		SNRNP200 E	-	-	
full-length	PRPF6	-		PRPF6 **	-	-	** 10mM 3-AT
full-length	CD2BP2	-		CD2BP2	-	-	
full-length	DDX23	-		DDX23	-	-	
full-length	WDR57	-		WDR57	-	-	
full-length	TXNL4A	-		TXNL4A **	-	-	** 10mM 3-AT
full-length	EFTUD2	-		EFTUD2	-	-	
full-length	PRPF19	-		PRPF19	-	-	

Positive control

ActII:	As2DD:
	EFTUD2
ZNHIT2	+
TSSC4	-
Alix (NC)	-

3-AT sensitivity test

	ActII:	As2DD:			
		SNRNP200 A	SNRNP200 D	TXNL4A	PRPF6
	RPAP3	+	+	+	+
	TSSC4	+	+	+	+
	Alix (NC)	+	+	+	+
+ 3-AT 2mM	RPAP3	+	+	+	+
	TSSC4	+	+	+	+
	Alix (NC)	+	+	+	+
+ 3-AT 5mM	RPAP3	-	-	+	+
	TSSC4	-	-	+	+
	Alix (NC)	-	-	+	+
+ 3-AT 10mM	RPAP3			-	-
	TSSC4			-	-
	Alix (NC)			-	-

Supplementary Table 2. A list of primers used in this study

Cloning primers

TSSC4	F: 5'-GGAAGATCTCATGGCTGAGGCAGGAACAGGTGAGCCG-3'<br>R: 5'-TCCCCGCGGGACCTCAGCACCTGGGTCCTC-3'
Δ51-100	F: 5'-GAGGGGGCGGCCAGACGG-3'<br>R: 5'-GGACAGTGCTTCCACTTCAGCACCAC-3'
Δ101-150	F: 5'-CCGGTCCCCGACTACGTG-3'<br>R: 5'-CAGGCAGTCAAAGATGTCACGG-3'
Δ151-200	F: 5'-TTCAACCAGGATCCCTCCAG-3'<br>R: 5'-AGGCACCCTTGGTGAGGC-3'
Δ201-250	F: 5'-GGCGAGGGCCCTGTGGAG-3'<br>R: 5'-GGAGGACACGCAGTCAGTGG-3'
Δ251-300	F: 5'-ACTGTTGGCTTCCATGGC-3'<br>R: 5'-CCTGTCTGTGGCAGGATTC-3'
ΔHom2	F: 5'-GAGGGGGCGGCCAGACGGGCT-3'<br>R: 5'-CGTGGCTGGGAGGAGGCCTGA-3'
ΔHom4	F: 5'-GTCCTGGGGAAGGTGGGAGAG-3'<br>R: 5'-GGACACGCAGTCAGTGGGGGC-3'
ΔHom2ΔHom3	F1: 5'-GAGGGGGCGGCCAGACGGGCT-3'<br>R1: 5'-CGTGGCTGGGAGGAGGCCTGA-3'<br>F2: 5'-GTCAGCGAGCAGAGCAATCAGGC-3'<br>R2: 5'-CACCCTTGGTGAGGCAGGGCT-3'
Hom1-1_Ala	F: 5'-GCTGCAGCCGCCCTCAGTGACTCGGACTCTGAC-3'<br>R: 5'-TGCAGCGGCCGTGTCATACTCCGTCCCGTGTT-3'
Hom1-2_Ala	F: 5'-GCGGCAGCTGCCCTCAGCTTGCCCGGTGGTGCT-3'<br>R: 5'-GGCAGCTGCGGAGACTGTGTCGGAAGGCAGCG-3'
Hom2-1_Ala	F: 5'-GCCGCCGCAGCCATGAGCTCCACCTTCTCCCAG-3'<br>R: 5'-AGCTGCAGCAGCCGTGGCTGGGAGGAGGCCTGA-3'
Hom2-2_Ala	F: 5'-GCAGCTGCAGCTAGCCGTGACATCTTTGACTGC-3'<br>R: 5'-AGCTGCAGCTGCGCCTCTCAGATGGAATGGCTG-3'
Hom2-3_Ala	F: 5'-GCAGCTGCAGCTGAGGGGGCGGCCAGACGGGCT-3'<br>R: 5'-GGCTGCGGCGGCGCGCTGGGAGAAGGTGGAGCT-3'
Hom4-1_Ala	F: 5'-GCAGCTGCATGTGGGGAGGGGAGGGTCATC-3'<br>R: 5'-TGCTGCAGCGAAGGAGGACACGCAGTCAGT-3'
Hom4-2_Ala	F: 5'-GCCGCGGCAGTCCGAGGGGTCGAAGCCAGA-3'<br>R: 5'-TGCTGCAGCCCTCCCCTCCCCACAGCTGGA-3'
Hom4-3_Ala	F: 5'-GCTGCTGCTAGGGTCCTGGGGAAGGTGGGA-3'<br>R: 5'-TGCTGCGGCTACGACCCCTCGGACTGG-3'
Hom2	F: 5'-ATATCTCGAGATGTCCCCGATGGGGCTG-3'<br>R: 5'-ATATGGTACCACCAGGCAGTCAAAGATGTCACG-3'
Hom2Hom3	F: 5'-ATATCTCGAGATGGCCACGGTGCAGCCATTC-3'<br>R: 5'-ATATGGTACCTTCTCGCTGACCTCGGTCACATC-3'
Hom4	F: 5'-ATATCTCGAGATGTTCAACCAGGATCCCTCCAGC-3'<br>R: 5'-ATATGGTACCCCCCTGTCTGTGGCAGGATTC-3'
Hom3Hom4	F: 5'-ATATCTCGAGATGCCTCCGGTCCCCG-3'<br>R: 5'-ATATGGTACCCCCCTGTCTGTGGCAGGATTC-3'
siRNA resistant TSSC4	F: 5'-GAGGTCGAGGCACTGTCCCCGATGGGGCT-3'<br>R: 5'-TGCGCCGCCAGGCAAGCTGAGGTCAGAGTC-3'

FISH probes

U2 snRNA	5'-[Cy3/A488]GAACAGATACTACACTTGATCTTAGCCAAAAGGCCGAGAAGC-3'
U4 snRNA	5'-[Cy3/A488]TCACGGCGGGGTATTGGGAAAAGTTTTCAATTAGCAATAATCGCGCCT-3'
U5 snRNA	5'-[Cy3/A488]CTCTCCACGGAAATCTTTAGTAAAAGGCGAAAGATTTATACGATTTGAAGAG-3'
U6 snRNA	5'-[Cy3/A488]CACGAATTTGCGTGTCATCCTTGCGCAGGGGCCATGCTAATC-3'

Northern blot probe

U5 snRNA	5'-TTGGGTTAAGACTCAGAGTTGTTCCTCTCC-3'

qPCR primers (for splicing efficiency analysis)

ACTB spliced	F: 5‘-CGTGCGTGACATTAAGGAGA-3‘<br>R: 5‘-ACAGGACTCCATGCCCAG-3‘
ACTB unspliced	F: 5‘-AGCTAAGTCCTGCCCTCATT-3‘<br>R: 5‘-GTACAGGTCTTTGCGGATGT-3‘
ALDOA spliced	F: 5‘-TATCAAATCCAAGGGCGGTG-3‘<br>R: 5‘-GCTCCGTCCTTCTTGTACTG-3‘
ALDOA unspliced	F: 5‘-TATCAAATCCAAGGGCGGTG-3‘<br>R: 5‘-ATTCCCTGCCTCACTAACCT-3‘
B2M spliced	F: 5‘-AGATGTCTCGCTCCGTGG-3‘<br>R: 5‘-CGTGAGTAAACCTGAATCTTTGG-3‘
B2M unspliced	F: 5‘-AGATGTCTCGCTCCGTGG-3‘<br>R: 5‘-CTTGGAGAAGGGAAGTCACG-3‘
GAPDH spliced	F: 5‘-ACATCGCTCAGACACCATGG-3‘<br>R: 5‘-GTTAAAAGCAGCCCTGGTGA-3‘
GAPDH unspliced	F: 5‘-CAGGGAAGCTCAAGGGAGAT-3‘<br>R: 5‘-GTTAAAAGCAGCCCTGGTGA-3‘
LDHA spliced	F: 5‘-TGGCAGCCTTTTCCTTAGAA-3‘<br>R: 5‘-CTTTCTCCCTCTTGCTGACG-3‘
LDHA unspliced	F: 5‘-TGGCAGCCTTTTCCTTAGAA-3‘<br>R: 5‘-TGTGCAACTGCACTCTACCC-3‘
PGK1 spliced	F: 5‘-ACAACCAGATAACAAACAACCAG-3‘<br>R: 5‘-GAGTACTTGTCAGGCATGGG-3‘
PGK1 unspliced	F: 5‘-TGTTGTCTCTCTTTGGTTGCA-3‘<br>R: 5‘-GAGTACTTGTCAGGCATGGG-3‘
RPL19 spliced	F: 5‘-ATGCCAGAGAAGGTCACATG-3‘<br>R: 5‘-CACATTCCCCTTCACCTTCA-3‘
RPL19 unspliced	F: 5‘-ATGCCAGAGAAGGTCACATG-3‘<br>R: 5‘-ACTAGCCATCAAAGCAGCAA-3‘
