# Supplementary material for: TSSC4 is a component of U5 snRNP that promotes tri-snRNP formation
Source: Nat Commun. 2021 Jun 15;12:3646. doi: 10.1038/s41467-021-23934-y (PMC8206348; doi:10.1038/s41467-021-23934-y)
Supplement: Supplementary file 3 — Description of Additional Supplementary Files [file 41467_2021_23934_MOESM3_ESM.pdf]

# Description of Additional Supplementary Files

File name: Supplementary Data 1

Description:  Characterization of proteins coprecipitating with TSSC4-GFP and TSSC4-FLAG by SILAC-IP. The ratio M/L shows a SILAC ratio of medium labeled proteins co-precipitated with tagged TSSC4 (M) and control immunoprecipitation from unlabeled cells (L).
